# Hematological Indices in Patients With Goiter: A Cross-Sectional Study in a Tertiary Care Hospital in South India

**DOI:** 10.7759/cureus.47227

**Published:** 2023-10-17

**Authors:** Kuzhalmozhi Manoharan, Karthik Sigamani, Muthulakshmi Vanniappan

**Affiliations:** 1 Pathology, Government Arignar Anna Memorial Cancer Hospital, Chengalpattu Medical College, Chengalpattu, IND; 2 Pathology, Karpaga Vinayaga Institute of Medical Sciences and Research Centre, Chengalpattu, IND; 3 Pathology, Arignar Anna Memorial Cancer Hospital, Chengalpattu Medical College, Tamil Nadu, IND

**Keywords:** euthyroid, hyperthyroidism, hypothyroidism, early diagnosis, anemia, total wbc count, packed cell volume, mean corpuscular volume, thyroid stimulating hormone, red cell distribution width

## Abstract

Introduction

A wide range of haematological abnormalities have been observed in patients with goitre. The aim of the study was to evaluate the abnormalities in haematological parameters among patients with goitre in a tertiary care hospital in south India.

Methods

This was a cross-sectional study carried out in the pathology department of Chengalpattu Medical College from April 1 to June 30, 2019. The lab reports, including the complete blood count (CBC) and serum thyroid profile that included thyroid-stimulating hormone (TSH), triiodothyronine (T3), and thyroxine (T4) of all the patients with goitre, were retrieved from the laboratory records. Results were tabulated and analysed.

Results

Out of a total of 200 patients with thyroid dysfunction, 12 (6%) were males and 188 (94%) were females, with the majority (51.5%) of them in the age group of 30-60 years. Serum TSH levels showed a statistically significant association with red cell distribution width (RCDW) (P-value = 0.000), mean corpuscular volume (MCV) (P-value = 0.020), and total white blood cell (WBC) count (P-value = 0.003) among the patients with goiter. There was no significant association between TSH and other parameters like haemoglobin, packed cell volume (PCV), red blood cell (RBC) count, and platelet (PLT) count.

Conclusions

Red cell distribution width and mean corpuscular volume are useful haematological parameters that will help clinicians in the early diagnosis and proper treatment of haematological abnormalities seen in patients with goitre.

## Introduction

The thyroid gland is one of the vital endocrine organs in the human body that secretes thyroid hormones. Thyroid hormones influence the normal development, physiological functions, and metabolic activity of almost all organ systems in our body [1]. The haematopoietic system is one of the primary systems influenced by thyroid hormones through several mechanisms. Thyroid hormones play an important role in regulating haematopoiesis in humans.

Thyroid-stimulating hormone (TSH), which is secreted by the anterior pituitary gland, mediates the output of thyroid hormones, namely triiodothyronine (T3) and thyroxine (T4), which in turn regulate erythropoiesis. All these functions are regulated by the binding of the T3 hormone to nuclear receptors [2]. Thyroid hormones stimulate erythropoiesis through their direct effect on bone marrow progenitor cells. In addition to this direct effect, it also plays an indirect role by regulating iron absorption, vitamin B12 absorption, and modulating erythropoietin production [3].

Thyroid dysfunction results in the enlargement of the thyroid gland, referred to as goitre. Goitre can be associated with a wide variety of haematological abnormalities, the most common being anaemia of different morphological types, namely microcytic hypochromic, macrocytic, and normocytic normochromic anaemia. Anaemia has been identified in 20-60% of hypothyroid patients, and several mechanisms are involved in its pathogenesis [4]. In every case of anaemia with an uncertain aetiology, the possibility of hypothyroidism should be considered.

In the laboratory evaluation of anaemia, haematological indices such as mean corpuscular volume (MCV), packed cell volume (PCV), haemoglobin (HB), red cell distribution width (RDW), and red blood cell (RBC) count are useful for diagnosis and categorising the morphological type of anaemia. Other parameters, like the total white blood cell (WBC) count and platelet count (PLT), also show variations among patients with thyroid dysfunction. Most of these haematological parameters have been found to be altered with a decline in thyroid function, namely hypothyroidism [5].

Hence, this study is aimed at evaluating the alteration in haematological parameters among patients with goitre in a tertiary care hospital.

## Materials and methods

Setting

This was a cross-sectional study carried out in the pathology department of Chengalpattu Medical College, Chengalpattu district, Tamil Nadu. The study period was three months, from April 1 to June 30, 2019.

Study population

Inclusion Criteria

The study included all patients with goitrous enlargement of the thyroid gland attending the central clinical laboratory of Chengalpattu Medical College. Blood samples from patients of all age groups and genders with goitre were collected.

Exclusion Criteria

Patients with other haematological conditions like iron deficiency anaemia and thalassemia; patients with systemic diseases, chronic renal diseases, and pregnant females; and patients on drug intake were not included in the study.

Sample size and sampling

A purposive sampling technique was used for the selection of desired samples according to the inclusion criteria. All the blood samples received from patients with goitre in the central clinical laboratory during the three-month study period were used for the study.

Protocol

The study was approved by the Chengalpattu Medical College, Institutional Ethical Committee for Human Studies (approval number: CMCH/19/PR/071). Informed consent was obtained from all patients during blood sample collection. This was a laboratory-based study where the reports, including the complete blood count (CBC) and serum thyroid profile that included TSH, T3, and T4, of all the blood samples from patients with goitre received from April 1 to June 30, 2019 in the central clinical laboratory were retrieved from the laboratory records. All the findings were tabulated and analysed. A TSH value of 0.4-5 IU/L was taken as a reference range for euthyroid status. Thus, patients with TSH values >5 IU/L were considered hypothyroid, while those with TSH values <0.4 IU/L were considered hyperthyroid cases. The RDW value of 12-14%, HB of 12-14 g, PCV of 34-36%, and RBC count of 4.5-5.5 million/cu.mm, MCV of 80-100 fL, WBC count of 4000-11,000/cu.mm, and platelet count of 1,50,000-4,00,000/cu.mm were taken as the normal reference range for the haematological parameters in this study.

Statistical analysis

Data were entered into Microsoft Excel (Microsoft® Corp., Redmond, WA) and analysed using Statistical Package for Social Sciences (SPSS) software version 22.0 (SPSS, Inc., Chicago, IL). Fisher’s exact test and Chi-square test were used to evaluate the association between TSH and complete blood count parameters. A P-value of 0.05 was taken as the cut-off point to determine statistically significant results. Frequency and percentage were calculated for categorical variables like patient demographic data (age, gender, etc.).

## Results

In the present study, a total of 200 patients who presented with goitre were included, out of which 188 (94%) were females and 12 (6%) were males. The majority of the patients (51.5%) were in the age group of 30-60 years (Table 1). The thyroid profile of all 200 patients revealed that 100 (50%) were euthyroid, 82 (41%) were hypothyroid, and 18 (9%) were hyperthyroid (Table 1). Among the haematological indices, the majority of the patients with goitre showed an alteration in MCV (82%), followed by RDW (54.5%) and RBC count (45%). The least affected RBC parameters were PCV (8.5%) and haemoglobin (3.5%). Thus, anaemia was seen in only 8.5% of the patients based on the reduction in PCV, which is a more sensitive haematological parameter compared to haemoglobin in the diagnosis of anaemia. However, other haematological indices like MCV, RDW, and RBC count showed alteration in the majority of patients with goitre even in the absence of anaemia. The total WBC count was altered in 56 (28%) cases, with one (0.5%) of them showing leukopenia, while the platelet count was altered in 23 (11.5%) cases, with two (1%) of them showing thrombocytopenia (Table 1).

**Table 1:** Frequency of various parameters among the patients with goiter TSH: thyroid-stimulating hormone; HB: haemoglobin; MCV: mean corpuscular volume; RDW: red cell distribution width; PCV: packed cell volume; RBC: red blood cell; WBC: white blood cell

Variables		Frequency	Percentage
Age group	<30 years	94	47%
	30-60 years	103	51.5%
	>60 years	3	1.5%
TSH	Normal	100	50%
	Hypothyroid	82	41%
	Hyperthyroid	18	9%
HB	Normal	193	96.5%
	Altered	7	3.5%
MCV	Normal	36	18%
	Altered	164	82%
RDW	Normal	91	45.5%
	Altered	109	54.5%
PCV	Normal	183	91.5%
	Altered	17	8.5%
RBC	Normal	110	55%
	Altered	90	45%
WBC	Normal	144	72%
	Altered	56	28%
Platelet	Normal	177	88.5%
	Altered	23	11.5%

In this study, all the patients, including both males and females, were categorised into three groups based on their TSH levels: euthyroid (0.4-5 IU/L), hypothyroid (> 5 IU/L), and hyperthyroid (<0.4 IU/L). Fisher’s exact test revealed a statistically insignificant association between the age groups and TSH levels among the three groups of patients in this study (P-value = 0.132). All the haematological parameters were compared with the TSH values among the three groups of patients and statistically analysed.

Among the 164 patients with altered MCV values, 54.3% were euthyroid, 36.6% were hypothyroid, and 9.1% were hyperthyroid. Among the total 36 patients with normal MCV values, the majority (61.1%) were hypothyroid. Fisher’s exact test revealed a statistically significant association (P-value = 0.020) between MCV and TSH levels in this study (Table 2).

**Table 2:** Association between TSH levels and other parameters *Statistically significant (P-value is significant at <0.05 TSH: thyroid-stimulating hormone; RDW: red cell distribution width; MCV: mean corpuscular volume; HB: haemoglobin; RBC: red blood cell; PCV: packed cell volume; WBC: white blood cell; PLT: platelet

Variables		TSH			Test value	P-value
		Euthyroid (100)	Hypothyroid (82)	Hyperthyroid (18)		
Age group	<30 years (94)	52 (53.3%)	32 (34%)	10 (10.6%)	Fisher’s exact test 6.422	0.132
	30-60 years (103)	47 (45.6%)	49 (47.6%)	7 (6.8%)		
	>60 years (3)	1 (33.3%)	1 (33.3%)	1 (33.3%)		
RDW	Normal (91)	72 (79.1%)	14 (15.4%)	5 (5.5%)	Chi square test 57.32	0.000*
	Increased (109)	28 (25.7%)	68 (62.4%)	13 (11.9%)		
MCV	Normal (36)	11 (30.6%)	22 (61.1%)	3 (8.3%)	Fisher’s exact test 7.849	0.020*
	Altered(164)	89 (54.3%)	60 (36.6%)	15 (9.1%)		
HB	Normal (193)	95 (49.2%)	80 (41.5%)	18 (9.3%)	Fisher’s exact test 0.920	0.611
	Altered (7)	5 (71.4%)	2 (28.6%)	0 (0%)		
RBC	Normal (110)	56 (50.9%)	45 (40.9%)	9 (8.2%)	Chi-square test 0.233	0.912
	Altered (90)	44 (48.9%)	37 (41.1%)	9 (10%)		
PCV	Normal (183)	88 (48.1%)	78 (42.6%)	17 (9.3%)	Fisher’s exact test 2.856	0.196
	Altered (17)	12 (70.6%)	4 (23.5%)	1 (5.9%)		
WBC count	Normal (144)	81 (56.3%)	55 (38.2%)	8 (5.6%)	Chi-square test 11.785	0.003*
	Altered (56)	19 (33.9%)	27 (48.2%)	10 (17.9%)		
PLT	Normal (177)	90 (50.8%)	72 (40.7%)	15 (8.5%)	Fisher’s exact test 1.012	0.601
	Altered (23)	10 (43.5%)	10 (43.5%)	3 (13%)		

Among the 109 patients with increased RDW values in the present study, the majority (62.4%) were hypothyroid, and among the total 91 patients with normal RDW values, the majority (79.1%) were euthyroid. The chi-square test revealed a highly significant association (P-value 0.000) between increased RDW values and TSH levels in this study (Table 2).

In the present study, other RBC haematological parameters like haemoglobin (P-value = 0.611, PCV-value = 0.196), and RBC count (P-value = 0.912) showed statistically insignificant association with TSH levels among the three groups of patients (Table 2).

In this study, among the 56 patients with an altered WBC count, 48.2% were hypothyroid, 33.9% were euthyroid, and 17.9% were hyperthyroid. Among the total 144 patients with a normal WBC count, the majority (56.3%) of them were euthyroid. The chi-square test revealed a statistically significant association (P-value = 0.003) between total WBC count and TSH levels in this study (Table 2).

In this study, among the 23 patients with an altered platelet count, 13% were hyperthyroid, 43.5% were euthyroid, and 43.5% were hypothyroid. Among the total 177 patients with a normal platelet count, the majority (50.8%) of them were euthyroid. There was no significant association (P-value = 0.601) between platelet count and TSH levels among the three groups of patients (Table 2).

## Discussion

The prevalence of goitre resulting from thyroid dysfunction is constantly increasing worldwide, especially in women compared to men. Thyroid dysfunction in the form of hypothyroidism or hyperthyroidism is associated with a wide range of haematological abnormalities, including pancytopenia, in many untreated cases [6]. Thyroid dysfunction is identified by measuring serum TSH levels and is now considered the most sensitive test among patients with goitre [7].

In this study, females accounted for 94% of the total patients with goitre, and the common age group was 30-60 years (51.5%). Iddah et al. [8] reported that 95% of female patients with thyroid dysfunction had a median age of 41 years, similar to the present study.

In the present study, anaemia was seen in 8.5% of patients, leukopenia in 0.5% of cases, and thrombocytopenia in 1% of cases. In the study by Iddah et al. [8], anaemia was encountered in 28.4% of cases, leukopenia was seen in 12.2% of cases, and thrombocytopenia was seen in 4.7% of cases, unlike the present study.

There was no significant association between the age groups and TSH levels among the three groups of patients in this study (P-value = 0.132), similar to the study by Geetha and Srikrishna [7]. Geetha and Srikrishna [7] reported a positive association (P-value < 0.001) between MCV and serum levels of TSH similar to the present study (P-value = 0.020). MCV reflects the size of RBCs. It is believed that thyroid dysfunction is associated with premature ageing of RBCs and increased lipolytic tendency of RBCs, along with altered distribution of lipids in the RBC’s membrane, thereby altering the MCV values [7]. MCV is increased in hypothyroidism and decreased in hyperthyroid patients.

RDW, which is used for quantitative measurement of variation in the size of RBCs, is calculated by dividing RBC SD by MCV. The RDW value is calculated routinely by automated haematology analyzers used to determine the CBC. In a study by Arundhathi et al. [9], RDW was significantly increased in hypothyroid patients, similar to the present study. Yu et al. [10] reported that RDW was significantly increased in patients with subclinical hypothyroidism and thus will help clinicians detect thyroid dysfunction at an early stage.

In the present study, there was no significant association between TSH levels and other RBC parameters like haemoglobin (P-value = 0.611), PCV (P-value = 0.196), and RBC count (P-value = 0.912), in concordance with the studies by Shouree et al. [6] and Geetha and Srikrishna [7]. In contrast to these observations, Dorgalaleh et al. [11] reported a statistically significant association between TSH levels and RBC count, PCV, RDW, and haemoglobin.

Yu et al. [10] and Bashir et al. [12] reported a statistically significant association between TSH levels and total WBC count similar to the present study (P-value = 0.003). However, Arundhathi et al. [9] reported no significant association between total WBC count and TSH levels. The mean WBC count was lower in hypothyroid patients compared to hyperthyroid patients in the study by Siddegowda et al. [13].

There was no significant association (P-value = 0.601) between platelet count and TSH levels in this study, similar to the studies by Shouree et al. [6] and Arundhathi et al. [9]. Platelet counts are less affected in thyroid dysfunction due to the fact that platelets are non-nucleated cells and have a shorter life span with rapid turnover [14].

Thus, among the various haematological parameters, the most significant and consistent association with derangement in TSH levels was shown by RDW and MCV in the majority of studies. However, RDW will be affected by other chronic medical conditions such as renal diseases, rheumatoid arthritis, and cardiac diseases [15].

Limitations

The present study was based on laboratory data. The clinical follow-up of the patients with goitre and the radiological features were not included in this study. Further studies incorporating the clinical, radiological, and laboratory data will provide better insight into the relationship between the various haematological parameters and the clinical severity of thyroid dysfunction.

## Conclusions

The clinical manifestations of thyroid dysfunction among patients with goitre typically develop slowly over a period of weeks to months. The most common manifestation of thyroid dysfunction includes haematological abnormalities, which, if not diagnosed and treated appropriately, can lead to serious life-threatening complications. CBC should be routinely done in the clinical evaluation of all patients with goiter. Among the various CBC parameters, RDW and MCV can serve as simple and cost-effective parameters for early diagnosis and appropriate management of patients with haematological abnormalities associated with sub-clinical hypothyroidism.
